# Fine mapping of the panicle length QTL *qPL5* in rice

**DOI:** 10.1007/s11032-024-01443-2

**Published:** 2024-01-17

**Authors:** Pengfei Wang, Ling Ma, Daoyang Li, Bo Zhang, Tianhao Zhou, Xiangchun Zhou, Yongzhong Xing

**Affiliations:** National Key Laboratory of Crop Genetic Improvement, Hubei Hongshan Laboratory, Huazhong Agricultural University, Wuhan, 430070 China

**Keywords:** aus, Panicle length, QTL, Fine mapping, Early mature

## Abstract

Panicle length is a crucial trait tightly associated with spikelets per panicle and grain yield in rice. To dissect the genetic basis of panicle length, a population of 161 recombinant inbred lines (RILs) was developed from the cross between an *aus* variety Chuan 7 (C7) and a tropical Geng variety Haoboka (HBK). C7 has a panicle length of 30 cm, 7 cm longer than that of HBK, and the panicle length was normally distributed in the RIL population. A total of six quantitative trait loci (QTLs) for panicle length were identified, and single QTLs explained the phenotypic variance from 4.9 to 18.1%. Among them, three QTLs were mapped to the regions harbored *sd1*, *DLT*, and *Ehd1*, respectively. To validate the genetic effect of a minor QTL *qPL5*, a near-isogenic F_2_ (NIF_2_) population segregated at *qPL5* was developed. Interestingly, panicle length displayed bimodal distribution, and heading date also exhibited significant variation in the NIF_2_ population. *qPL5* accounted for 66.5% of the panicle length variance. The C7 allele at *qPL5* increased panicle length by 2.4 cm and promoted heading date by 5 days. Finally, *qPL5* was narrowed down to an 80-kb region flanked by markers M2197 and M2205 using a large NIF_2_ population of 7600 plants. LOC_Os05g37540, encoding a phytochrome signal protein whose homolog in *Arabidopsis* enlarges panicle length, is regarded as the candidate gene because a single-nucleotide mutation (C1099T) caused a premature stop codon in HBK. The characterization of *qPL5* with enlarging panicle length but promoting heading date makes its great value in breeding early mature varieties without yield penalty in rice.

**Supplementary Information:**

The online version contains supplementary material available at 10.1007/s11032-024-01443-2.

## Introduction

Rice is not only a staple food in Asia but also a crucial model crop for biological research. Rice yield is mainly contributed by spikelets per panicle. Panicle length, a crucial trait controlling rice panicle architecture, exerts a substantial influence on spikelets per panicle and yield per plant (Bai et al. 2016). Panicle length is coordinated by a complex gene cluster associated with panicle development, plant morphology, and heading date. Importantly, these genes shape panicle length via different molecular mechanisms.

In the past two decades, a series of important genes controlling panicle length have been identified. *APO1* encodes an F-box protein, and its genetic mutations cause premature cessation of meristem proliferation, culminating in shortened panicle length, which concurrently impacts both the number of branches and yield (Ikeda et al. 2005). *APO2*, like the Arabidopsis gene *FLY*, functions as a homologous counterpart to hinder the meristematic transition from inflorescences to spikelets, while the loss of *APO2* function results in reduced panicle lengths and branches (Rao et al. 2008). LARGE2 interacts with both APO1 and APO2 and negatively modulates the stability of these proteins, while loss of *LARGE2* function leads to the accumulation of APO1 and APO2, thereby yielding enlarged panicle architecture (Huang et al. 2021). SP1 acts as a polypeptide transporter and affects panicle length (Li et al. 2009). *DEP1* encodes the γ subunit of a trimeric G protein, while loss of *DEP1* functions influences both panicle length and grain density (Huang et al. 2009). The ideal plant architecture determinant gene, *IPA1*, positively regulates *DEP1*, resulting in substantial alterations in panicle length (Miura et al. 2010; Jiao et al. 2010). Furthermore, genes such as *sd1*, *Ghd7*, *Ghd7.1*, and *Ghd8*, which show significant effects on plant architecture or heading date, also exhibit a minor impact on panicle length (Sasaki et al. 2002; Xue et al. 2008; Yan et al. 2013; Yan et al. 2011). The previous studies are mainly focused on the populations derived from limited rice subgroups, which exhibit a broader geographical distribution and wider production application.

Asian cultivated rice has evolved into two subspecies: Xian/*indica* and Geng/*japonica* (Ting 1949). Generally, Xian varieties exhibit longer panicle length than Geng varieties. The two subspecies are further categorized into multiple subgroups, such as East Asian Xian, IRRI Xian, South Asia Xian, Southeast Asia Xian, *aus*, Temperate Geng, Tropical Geng, and Basmati, demarcated by morphological characteristics, geographical distribution, and pedigree connections (Wang et al. 2018). Over the past two decades, numerous studies have explored the genetic basis of quantitative traits in rice. But the *aus* rice, which is regarded as a valuable resource for mining favorable genes/alleles (Casartelli et al. 2018), has not been paid enough attention in QTL mapping.

*Aus* rice, primarily cultivated in challenging environment in Bangladesh and India, has undergone significant evolutionary adaptation due to prolonged natural selection and artificial domestication (Londo et al. 2006). This has conferred remarkable adaptability to environmental stress and unproductive conditions upon *aus* varieties. Notably, the *aus* variety N22 stands out as one of the most thermotolerant rice cultivars (Li et al. 2015). Moreover, the *aus* subgroup has contributed valuable genetic resources, including the flood tolerance gene *Sub1* and the phosphorus starvation tolerance gene *OsPSTOL1* (Xu et al. 2006; Gamuyao et al. 2012). The *aus* variety Dular contains wide compatibility alleles like a bridge connecting Xian and Geng subspecies, which accelerates the advancement of inter-subspecies hybridization breeding programs (Wang et al. 1998). The *aus* genetic resources harbor numerous unique genetic resources distinct from the conventional Xian and Geng varieties, while the exploration and recognition of these noteworthy genetic resources hold substantial promise for molecular breeding.

In this study, we developed a RIL population through the cross between the *aus* variety C7 with long panicles and the tropical Geng variety HBK with short panicles. The population was genotyped by resequencing, and a comprehensive genome-wide bin map was constructed. The genetic basis of panicle length was dissected, and a total of 6 QTLs were identified. To validate and fine map the QTL *qPL5* with a moderate effect, a near-isogenic F_2_ population was developed. *qPL5* was finally narrowed down to an interval of 80-kb region. The favorable allele from the *aus* variety exhibits valuable potential for breeding early mature varieties without yield penalty in rice.

## Results

### High polymorphism between the parents

**Fig. 1:**
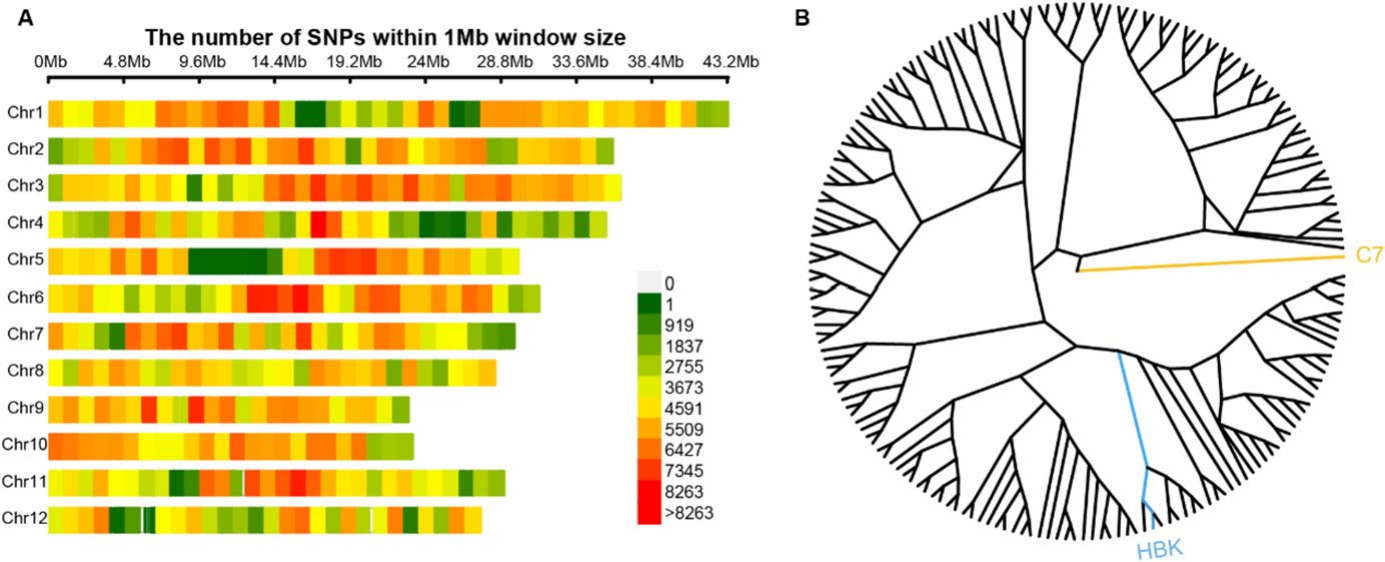
Distribution of SNPs on chromosomes and phylogenetic tree in the RIL population. **A** Distribution of SNPs within 1-Mb interval on 12 chromosomes. **B** Neighbor-joining tree of the two parents and 161 RILs, the two parents C7 and HBK are marked

The sequencing depth of two parents was exceeded 30 ×, and more than 2 G of high-quality resequencing data were generated for each of 161 RILs. Following stringent quality control, a total of 1,711,474 high-quality SNPs were identified (Fig. 1A). These variants exhibited a distinct distribution across chromosomes. Chromosome 10 exhibited the highest SNP density of 5.3 SNPs/kb, while chromosome 4 displayed the lowest density of 3.6 SNPs/kb. Approximately 2.7% of the identified SNPs were heterozygous across the population, slightly lower than the expected frequency (3.2%) for the F_6_ generation. A neighbor-joining phylogenetic tree constructed by 1354 evenly distributed SNPs with low linkage disequilibrium (LD) (*r*^2^ ≤ 0.2) revealed that the RIL population has no distinct population structure (Fig. 1B).

### Construction of high-density whole-genome bin map in the RIL population

**Fig. 2:**
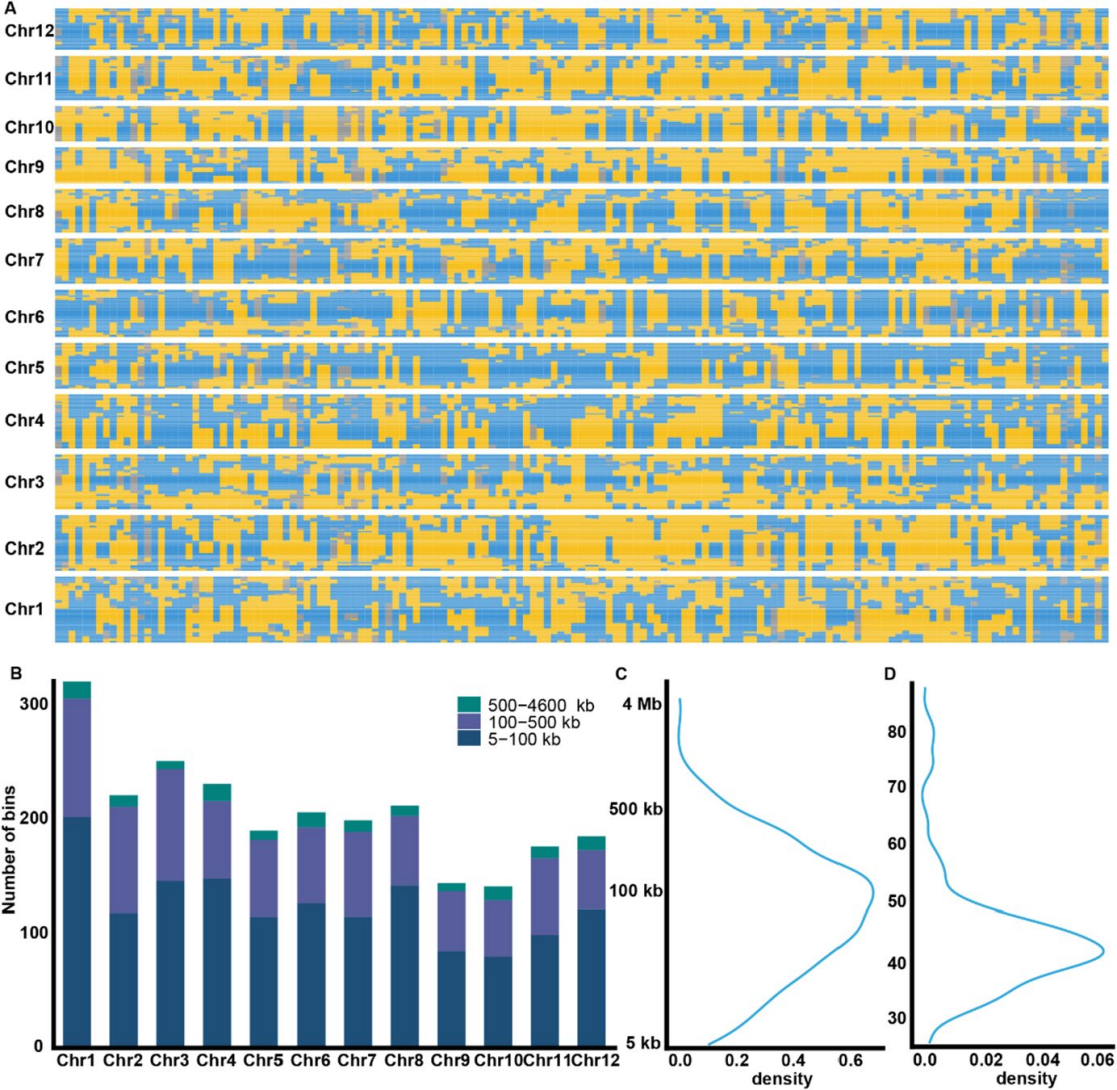
Whole-genome bin construction in RIL population. **A** The bin map of the RIL population, each column represents a RIL, blue is HBK, yellow is C7, and gray is heterozygous. **B** Distribution of bin length across 12 chromosomes at population level. **C** Density plot of bin length at population level, the *y* axis is the length of bin. **D** Density plot of bin number at individual level, the *y* axis is the number of bins per individual

**Fig. 3:**
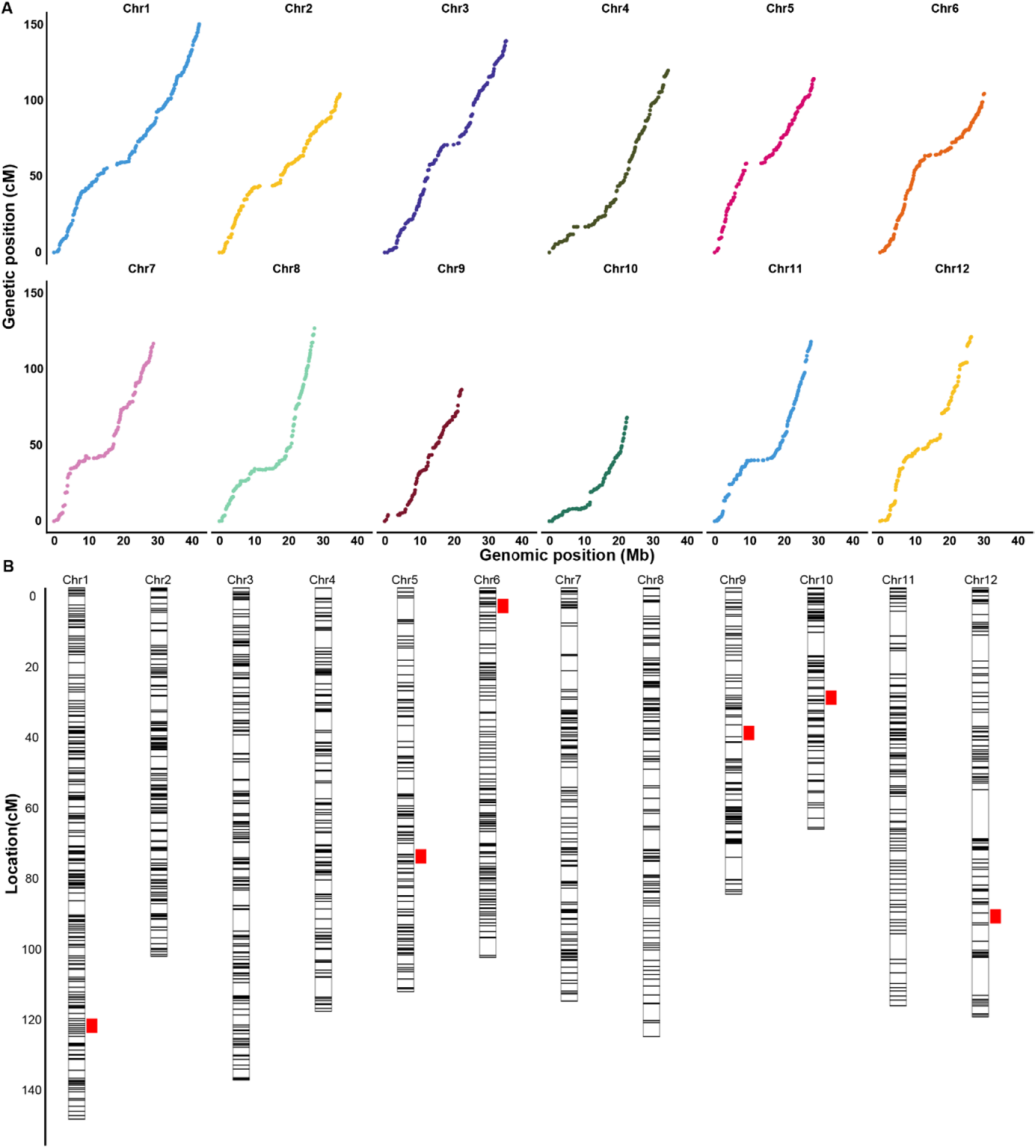
Collinearity analysis and genetic linkage map with the RIL population. **A** Collinearity analysis of genetic linkage map and physical map. **B** The genetic map showing QTLs for panicle length, black line represents each bin. Red box is the confidence interval of QTLs

A total of 2477 bins were identified in the RIL population (Fig. 2A), which is 1.5-fold to that detected in a larger RIL population with 197 individuals directly derived from F_1_ plants (Tan et al. 2013). This indicated the strategy constructing RIL by intercrossing F_2_ plants has increased the number of meiosis, resulting in a high-resolution map. At the population level, the bin lengths ranged from 5 to 4597 kb, with an average length of 150 kb (Fig. 2C). Most bins are smaller than 500 kb in length, while 60.4% of the bins are smaller than 100 kb (Fig. 2B). The longest bin was located on the centromeric region of chromosome 2 with a length of 4.6 Mb. Remarkably, the number of bins at the individual plants ranged from 25 to 88, with an average of 44 bins per line (Fig. 2D). The genetic composition of the RIL population included 49.2% homozygous *aus* genome, 46.7% homozygous Geng genome, and 4.1% heterozygous regions. On average, there were 7 recombinant events per Mb, ranging from 1 to 23. Recombination repression was observed in the centromeric regions of all 12 chromosomes, and one recombination hotspot was detected on chromosome 8 (sup Table 1). The genetic linkage map was developed covering 1397-cM regions, and genetic distance between adjacent bins was approximately 0.56 cM. The markers’ position in the physical map aligned very well with those in the genetic map (Fig. 3A).

### QTLs for panicle length

The panicle lengths of the two parents C7 and HBK were 30 cm and 23 cm, respectively. Panicle length exhibited a normal distribution ranged from 17.2 to 39.5 cm with a mean of 26.4 cm in the RIL population (sup Fig 1). Linkage analysis based on the ICIM model identified 6 QTLs on chromosomes 1, 5, 6, 9, 10, and 12 (Fig. 3B; Table 1). The major QTL, *qPL1* with an additive effect of 1.6 cm, accounted for 18.1% of the phenotypic variance and the C7 allele increasing panicle length. Its confidence interval harbored the well-documented major gene, *sd1*, known for its impact on plant architecture and panicle length (Sasaki et al. 2002). The minor QTL, *qPL6*, explained 8.0% of the phenotypic variance, and the HBK allele increased panicle length by 1.0 cm. The gene *DLT*, which affected the internode length and panicle length by modulating BR level, was located in the confidence interval (Tong et al. 2009). The confidence interval for another minor QTL, *qPL10*, contained *Ehd1* with an additive effect of 0.8 cm, which was reported to affect heading date and panicle length (Hu et al. 2019). Previously reported functional polymorphisms in the three genes were identified between two parents (Table 2). The missense variant in *sd1* coding region resulted in lower gibberellin biosynthetic activity and a shorter culm (Asano et al. 2011). The 5’ UTR variant in *DLT* affected its expression level, and *DLT* modulated panicle length by regulating gene expression of BR synthesis (Sun et al. 2013; Tong et al. 2009). The 3’ UTR variant in *Ehd1* was reported as its causal mutation (Wei et al. 2021). Therefore, these three genes are very likely underlying the three QTLs. The minor QTL, *qPL5* with an additive effect of 1.0 cm, accounted for 6.6% of the phenotypic variance, and the C7 allele increased panicle length. A previously characterized gene, *EUI1*, in the confidence interval of *qPL5* was documented for its role in influencing panicle length (Zhu et al. 2006).

**Table 1:** Putative QTLs for panicle length detected in the RIL population

QTL	Chr	Start	End	LOD	PVE %	Add
*qPL1*	1	38019364	38610747	11.2	18.1	1.6
*qPL5*	5	19403806	20167654	4.4	6.6	1.0
*qPL6*	6	1347846	2163965	5.3	8.0	−1.0
*qPL9*	9	12220801	14075636	5.5	8.6	1.1
*qPL10*	10	16069736	17494721	3.4	4.9	0.8
*qPL12*	12	21736638	22964216	4.7	7.0	1.0

*LOD*, logarithm of odds. *Add*, the additive effect. Positive values indicate that alleles from C7 increase the trait scores. *PVE*, phenotypic variance explained

**Table 2:** Functional polymorphisms between parents in known genes located in three QTL regions

QTL	Gene	Chr	Pos	C7	HBK	Anno	Reference
*qPL1*	*sd1*	Chr1	38385057	G	A	missense	Asano et al. (2011)
*qPL6*	*DLT*	Chr6	1468526	TCTCTCACTCTCA	T	5_prime_UTR	Tong et al. (2009)
*qPL10*	*Ehd1*	Chr10	17076177	C	T	3_prime_UTR	Wei et al. (2021)

*Anno*, the functional annotation of the variant

### Validation and fine mapping of *qPL5*

**Fig. 4:**
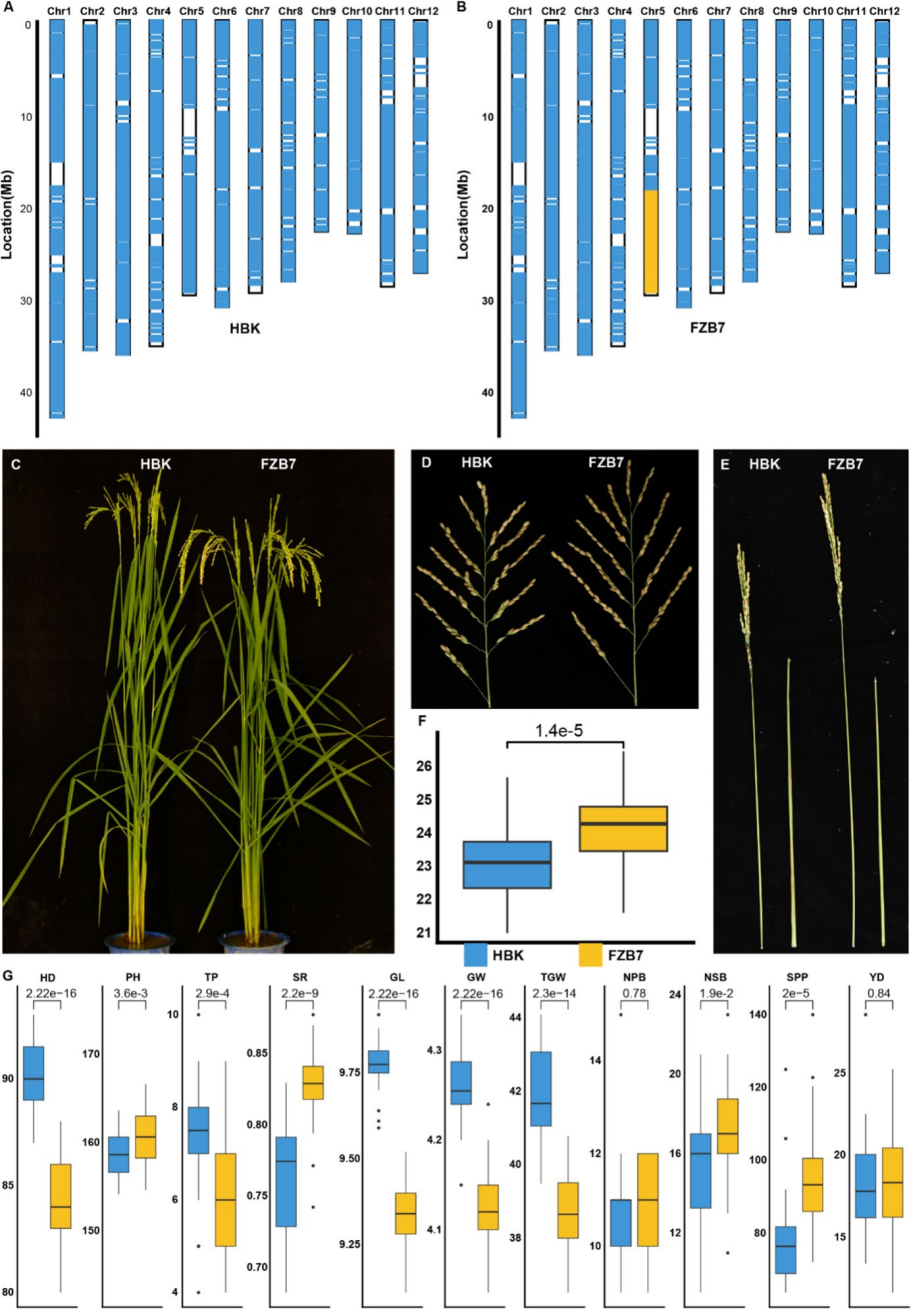
Genome constitution of FZB7 and phenotypic comparison between *qPL5* near-isogenic lines. **A** Genome constitution of HBK (**A**) and FZB7 (**B**) identified by rice 40K SNP array, blue is HBK genotype, white is the genome not covered by SNP markers, and yellow is C7 genotype. The comparison of whole plant (**C**), panicle architecture (**D**), internode length (**E**), and panicle length (cm) (**F**) between HBK and FZB7. **G** The comparisons of agronomic traits between HBK and FZB7. HD, heading date; PH, plant height (cm), TP, tillers per plant; SR, seed setting rate; GL, grain length (mm), GW, grain width (mm); TGW, thousand-grain weight (g); NPB, the number of primary branches; NSB, the number of second branches; SPP, spikelets per panicle; YD, yield per plant (g)

To validate the genetic effect of *qPL5*, we constructed a single-segment introgression line, FZB7, against the HBK background (Fig. 4A, B). Analysis of rice 40 K SNP array indicated a background recovery rate of 97.1%. Subsequent phenotypic evaluations revealed that the growth and developmental stages of FZB7 were nearly same as that of HBK (Fig. 4C). FZB7 exhibited a longer panicle length and an increased number of second branches (Fig. 4F, G), indicating the presence of the *qPL5* effect in the introgression fragment. Interestingly, significant increases were observed in plant height, seed setting rate, and spikelets per panicle in FZB7, while HBK showed a 5-day delayed heading date, 1 more tiller per plant, and a heavier thousand-grain weight (Fig. 4D, E, G). Consequently, no difference in yield per plant was detected between two genotypes. To exclude the possibility of *EUI1* as the candidate, Sanger sequencing was performed for *EUI1* in both parents. No variations in the *EUI1* coding region were detected between the parents (sup Data 1). Simultaneously, the expression patterns of *EUI1* were conducted in FZB7 and its recurrent parent HBK. No significant differences in expression pattern were ascertained (sup Fig 2). Therefore, *EUI1* was unlikely the causal gene for *qPL5*.

**Fig. 5:**
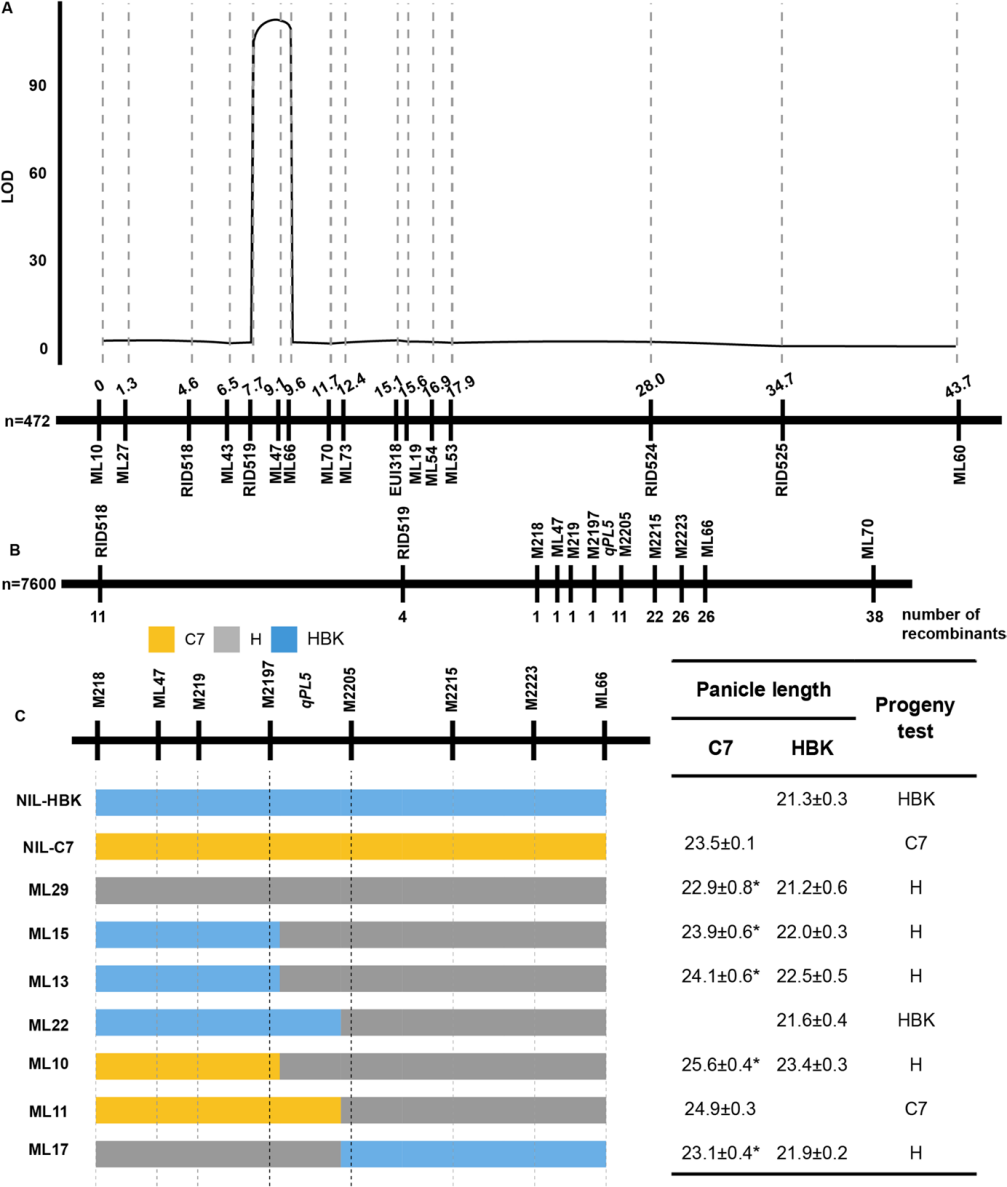
Fine mapping of *qPL5*. **A** QTL scanning for panicle length across the target region on chromosome 5 in the BC_4_F_2_ population. *X* axis is the genetic position, and *Y* axis is the LOD score. **B** The number of recombinants between *qPL5* and molecular markers in the BC_4_F_3_ population. **C** Progeny test of the recombinants. *, *P < 0.05*. More than 10 plants each two homozygous were used for comparison in the progeny test

To figure out the candidate gene, a BC_4_F_2_ population of 472 individuals was then generated through the cross between HBK and FZB7. The panicle length distributed with bimodal model ranging from 19.5 to 25.6 cm, and a large variation ranging from 90 to 105 days was identified in heading date in the BC_4_F_2_ population (sup Fig 3). To facilitate the fine mapping work, five polymorphic InDel markers and 11 polymorphic KASP markers were developed. Finally, *qPL5* was mapped to a 2.3-Mb interval delimited by markers RID518 and ML70 (Fig. 5A). The logarithm of odds (LOD) score attributed to *qPL5* showed a remarkable elevation to 112.2. Its additive effect on panicle length was 1.2 cm; it accounted for 66.5% of the panicle length variance (Table 3). Moreover, *qPL5* had effects on heading date and spikelets per panicle but no effects on yield per plant (Table 3).

**Table 3:** Pleiotropic effects of *qPL5* on panicle length, heading date, and spikelets per panicle in the NIF_2_ population

Trait	QTL	Confident interval	LOD	PVE %	Add	Dom
PL	*qPL5*	RID519-ML47	112.2	66.5	1.2	0.5
HD			132.5	72.3	−2.7	1.1
SPP			95.4	52.1	12.6	4.2

*Add*, additive effect; *Dom*, dominant effect

To further fine map *qPL5*, a BC_4_F_3_ population of 7600 plants was developed. From the large population, we screened 11 and 38 recombinants between *qPL5* and two markers RID518 and ML70, respectively (Fig. 5B). Additionally, 6 polymorphic InDel markers were developed in the target region between the markers RID518 and ML70. After progeny test of 15 recombinants screened by markers RID519 and M2205, *qPL5* was mapped to an 80-kb interval delimited by markers M2197 and M2205 (Fig. 5C), in which *EUI1* was not located.

### Possible candidate gene for *qPL5*

There were a total of 16 genes in the targeted 80-kb region in RGI database (Table 4) (Yu et al. 2023). Among them, the gene LOC_Os05g37540 was annotated to encode the protein of Far-red impaired Response 1 (FAR1), an essential transcriptional factor for phytochrome A signaling. In *Arabidopsis*, FAR1 directly interacts with three Squamosa-promoter binding protein-like (SPL) transcription factors, SPL3, SPL4, and SPL5, and inhibits their binding to the promoter of key flowering gene *FUL*, *LFY*, *AP1*, and *MIR172C*, thereby delaying flowering and inducing the development of enlarged panicles (Xie et al. 2020). In rice, mutations in phytochrome genes have been associated with a moderate acceleration of flowering under long day conditions (Su et al. 2016). A mutation C1099T was identified in the LOC_Os05g37540 exon of HBK, leading to a premature termination. The mutation was infrequently detected in tropical Geng rice based on the ricevarmap database (Zhao et al. 2021). Thus, LOC_Os05g37540 is a possible candidate gene for *qPL5*.

**Table 4:** Candidate genes in the 80-kb target region of *qPL5*

Gene	Annotation	C7	HBK
LOC_Os05g37530	Expressed protein	-	-
LOC_Os05g37540	Protein FAR1-RELATED SEQUENCE 5	-	StGV
LOC_Os05g37550	Expressed protein	-	-
LOC_Os05g37560	Expressed protein	StGV, MsV	-
LOC_Os05g37570	Putative; Ty3-gypsy subclass	-	-
LOC_Os05g37580	Putative; unclassified; expressed	-	-
LOC_Os05g37590	Transposon Ty3-I Gag-Pol polyprotein	-	-
LOC_Os05g37600	Glycerol-3-phosphate 2-O-acyltransferase 6	-	-
LOC_Os05g37610	Expressed protein	-	-
LOC_Os05g37620	Retrovirus-related Pol polyprotein from transposon RE1	StGV, MsV	-
LOC_Os05g37630	Expressed protein	MsV	-
LOC_Os05g37640	AP2/ERF transcription factor	-	-
LOC_Os05g37650	Expressed protein	MsV	-
LOC_Os05g37660	Uncharacterized acetyltransferase	MsV	-
LOC_Os05g37670	Putative; unclassified; expressed	StGV, MsV	-
LOC_Os05g37680	Putative; unclassified; expressed	FsV, MsV	-

*StGV*, stop gained variant; *MsV*, missense variant; *FsV*, frameshift variant

## Discussion

### LOC_Os05g37540 encoding a FAR1-related protein is a strong candidate gene of *qPL5*

In this study, *qPL5* was validated in a NIF_2_ population and fine mapped to an 80-kb region flanked by markers M2197 and M2205. There are 16 genes in the target region. The functional annotation highlighted three genes associated with plant development as potential candidates underlying *qPL5*. LOC_Os05g37600 encodes a member of the glycerol-3-phosphate acyltransferase family, which has been previously reported to be related to cellular metabolism associated with plant growth and development (Safder et al. 2021). LOC_Os05g37640 encodes an AP2/ERF transcription factor, while the AP2 transcription factors FZP, OsSNB, OsIDS1, and MFS1 were reported to affect panicle architecture (Ren et al. 2013; Lee and An 2012; Bai et al. 2017). But both genes have no functional polymorphism between parents (Table 4); they are excluded for the candidate. LOC_Os05g37540 encodes a FAR1-related protein. *FAR1* in *Arabidopsis* was associated with delayed flowering, which led to enlarged panicles (Xie et al. 2020). Moreover, *qPL5* has effects on heading date (Fig. 4G). More important is that there is a premature termination variation in LOC_Os05g37540 gene between the parents (sup Data 2). Taken together, LOC_Os05g37540 is strongly suggested as the candidate gene of *qPL5*, which will be eventually confirmed by gene transformation.

### Minor QTL is the target for gene cloning in the future

A total of six QTLs for panicle length were identified in the RIL population. Among these QTLs, only one QTL *qPL1* explained 18.1% of panicle length variance, which would be classified as a major QTL. All the other 5 QTLs are minor ones, each explaining less than 9% of the variance. Three genes, *sd1*, *DLT*, and *Ehd1*, which regulate panicle length by influencing GA biosynthesis, BR levels, and heading date, respectively (Sasaki et al. 2002; Tong et al. 2009; Hu et al. 2019), were located in the QTL regions of *qPL1*, *qPL6*, and *qPL10*, respectively. Moreover, the previously reported functional polymorphisms such as missense mutation and UTR variants were detected between the two parents (Table 2), strongly suggesting they are the genes underlying these three QTLs. In addition, the minor QTL *qPL5* was validated in a near-isogenic F_2_ population. The other two QTLs, *qPL9* and *qPL12*, which exhibited comparable effects to *qPL5*, were detected in several studies (Cui et al. 2002; Kobayashi et al. 2003; Xiao et al. 1998). Thus, the minor QTLs identified in this study are reliable. A rapid QTL cloning strategy by generating a pseudo-near-isogenic F_2_ population can be used to speed up the fine mapping of *qPL9* and *qPL12* without continuous advanced backcross (Sherif et al. 2023).

### *qPL5* is a valuable gene for breeding early mature varieties without yield penalty in rice

The *aus* rice is a valuable resource for mining favorable alleles because many superior alleles have been specially identified in *aus* subpopulation (Casartelli et al. 2018). The superior alleles at five of six QTLs for panicle length detected in this study were contributed by the *aus* cultivar C7, which is in accordance with the fact that C7 has the panicle length of 7 cm longer than HBK. The semidwarf gene *sd1* and *DLT* increase panicle length but significantly increase plant height which leads to lodging and substantial yield loss. *Ehd1* enlarges the panicle length by delaying heading, which influences the adaptation of varieties to cropping seasons and ecological zones (Hu et al. 2019). In contrast, *qPL5* showed negative effects on heading date and tiller number per plant and positive effects on plant height and seed setting ratio in the near-isogenic lines (Table 3). Ultimately, it has no significant impact on the yield per plant. Therefore, the *aus* C7 *qPL5* is a potential favorable allele for breeding early mature varieties without yield penalty. The closely linked markers M2197 and M2205 would be used for assistant selection of *qPL5*.

## Methods

### Plant materials

We developed a RIL population between an *Oryza sativa* *aus* inbred line (Chuan 7, C7) and an *Oryza sativa Geng* inbred line (Haoboka, HBK). The hybrid F_1_ plant was obtained by crossing C7 with HBK, and then several hundred F_2_ plants were obtained by self-crossing. From these, 16 F_2_ plants were randomly selected to make eight pairwise crosses. Eight hybrids from each pairwise cross were used for selfing, and then 3 individuals each self-bred family were randomly selected for continuous self-pollination for 5 generations by single seed descent (sup Fig 4). Finally, a total of 161 RILs were constructed. SSR markers were employed to confirm the true hybrid identity throughout the process.

A set of CSSLs (BC_4_F_1_) were previously developed with HBK as the recurrent parents and C7 as the donor parent (Zhang et al. 2021). A single-segment introgression line FZB7 possessed the *qPL5* was used to generate a BC_4_F_2_ population of 472 plants. A BC_4_F_3_ population of 7600 plants was used for fine map of *qPL5*. The progenies of the recombinants between markers and *qPL5* were tested in the field.

### Field experiments and panicle length measurement

Field experiments were performed at the experimental farm of Huazhong Agricultural University in Wuhan. The RIL population were sowed on May 18, 2018. Fourteen 25-day old seedlings each RIL were transplanted into a two-row plot, with a distance of 16.5 cm between plants within a row and 26.5 cm between rows. All plants except marginal and abnormal individuals were used to measure the panicle length, and 3 panicles were recorded for each plant. The average panicle length was used for QTL mapping. Four hundred seventy-two BC_4_F_2_ plants was planted on May 18, 2020. Seven thousand six hundred BC_4_F_3_ plants and progeny test for recombinants were sowed on May 21, 2021, and May 19, 2022, respectively.

### DNA extraction and re-sequencing

DNA was extracted from young leaves by the CTAB method. A DNA library was constructed using the NGS pipeline, and then paired-end sequencing was performed on the HiSeq 2000 platform in GENOSEQ (Wuhan, China) with a read length of 150 bp. The sequencing depth for the two parents and each RIL was 30× and 5×.

### Identification of DNA variations

The re-sequencing raw reads were trimmed and clipped the high-quality part of read by Fastp software (Chen et al. 2018). Then, clean reads were aligned to the Nipponbare reference genome from the Rice Genome Annotation Project (http://rice.uga.edu/), and Samtools was used to sort and convert the SAM file to BAM file (Danecek et al. 2021). PCR duplicates were removed by the MarkDuplicates program in the Picard software (https://broadinstitute.github.io/picard/). SNPs and InDels were called by the HaplotypeCaller and GenotypeGVCFs program with GVCF mode in the Genome Analysis Toolkit (McKenna et al. 2010). SNPs with missing rates above 50% and minor allele frequency above 10% were used for downstream analysis.

### Phylogenetic analysis

Independent SNPs were extracted using the independent pairwise method in R/SNPRelate package, employing a linkage disequilibrium threshold of 0.2 (Zheng et al. 2012). The distance matrix was calculated, and a phylogenetic tree was constructed by SNPRelate package. The ape and ggtree packages were used to display the tree and to label the two parents (Paradis and Schliep 2019; Yu et al. 2017).

### Bin map construction

Using the python program SNPbinner, bin map was conducted for the RIL population based on the recalibrated SNPs genotyped by the GATK pipeline to further decrease the impact of sequencing errors and computational consumption (Gonda et al. 2019).

### QTL mapping

Genetic map was constructed using R package Asmap with Kosambi mapping function (Kosambi 1943; Taylor and Butler 2017). QTL mapping was performed by the QTL IciMmapping software (Meng et al. 2015). Additive QTL was identified by inclusive composite interval mapping (ICIM) method. The threshold of the logarithm of odds (LOD) value was 2.5.

### Marker development

All markers for fine mapping were designed based on Nipponbare genome and parental differential variation. KASP mark was designed by snpway website (http://www.snpway.com/). All markers used in this experiment were listed in the Supplementary Table 2.

### Supplementary information

ESM 1

ESM 2

ESM 3

ESM 4

## Data Availability

All raw reads generated for the individuals in the study have been deposited in the National Genomics Data Center under BioProject PRJCA022700.
