# Supplementary material for: Fine mapping of the panicle length QTL qPL5 in rice
Source: Mol Breed. 2024 Jan 17;44(2):6. doi: 10.1007/s11032-024-01443-2 (PMC10794681; doi:10.1007/s11032-024-01443-2)
Supplement: Supplementary file 2 — ESM 2 [file 11032_2024_1443_MOESM2_ESM.docx]

>LOC_Os05g37540 NIP

ATGGATAAACAAGTGAAGAACCACAGACCTTCTGATGGTACGGATGGATCAAGGGAAATTCTTATGGACGAGCGCAACCCTACCATGGACATAGTAGATGGTATTAGTCAATCTGTCCATGATTCACAACCGGATGGTGGTCAGTCAAGCCGTGTAGCAATGGAGGAGGACGTGCCGTCGCGGGTTGGATCACATGATTCAGGGCATGGAATTCCAGAGACAAGAAATTTGGTCCCTTCACTGGATTGGACAGAAGGAAACCCATATGAAAAAGAAGGAGCTGGGATACCGATGGAAAGAATGGTGGTAGATAATGTTGATAGCAGTGCTGTCATACCTCCTGAGTATGCTGATCCTCAAATTCTTACTCCGATGCTGGGACAAAGATTTAAGACTGAGAGGGATGCCTATAATTTTTATAATGTTTATGCTGTTAGTAAAGGATTTGGGATACGACTGGACAAAGATCGCATGAATACTAAAAAGCAGAGAACAATGCGGCAAATATGTTGTTCTCACCAGGGGAGGAACCCAAAAACCAAGAAGCCTTCCGTTCGAATTGGGTGCCCAGCAATGATGAAGATAAATATGTCTGGAGCTGGTAGTGGCTGGTCAGTAACAAAGGTTGTTTCTGCGCACAACCATCCTATGAAAAAAAGTGTAGGAGTTACTAAGAATTATCAGTCGCACAATCAAATAGATGAGGGGACTCGTGGTATTATTGAAGAGATGGTAGACAGTAGTATGAGTCTTACAAATATGTATGGTATGTTGTCTGGGATGCATGGCGGACCTTCCATGGTTCCATTCACGAGAAAAGCTATGGATAGGGTTGCTTATGCAATTAGGCGGGATGAGAGCAGCGATGACATGCAAAAAACACTTGATGTCTTGAAGGATTTGCAAAAAAGGAGCAAGAATTTCTTTTATAGTATACAAGTTGACGAGGCTTGCCGTGTGAAGAACATATTTTGGTCTCATGCTGTGTCCAGATTGAACTTTGAGCATTTTGGTGATGTCATCACATTTGATACTACCTACAAAACAAACAAGTATAATATGCCCTTTGCACCATTTGTTGGTGTAAACAATCACTTTCAGAGCACCTTCTTTGGTTGTGCCCTGCTTAGAGAAGAGACTGAAGAATCATTTACATGGTTATTCAATACATTCAAAGAGTGCATGAATGGGAAGGTTCCTATTGGAATATTAACAGACAATTGTCCTTCCATGGCAGCTGCCATTAGAACAGTATTTCCCAATACAATCCATCGTGTGTGCAAGTGGCATGTACTGAAGAAAGCAAAGGAATTTATGGGGAACATATACTCCAAGCGTCATACATTCAAGAAAGCATTTCATAAGGTTCTTTGCTGGGATGACTACTACACAGAGAACTCTATGAATGGCTTTGTTAAGAGGTACGACCGGTTTTTCAATGAGAAGCTGCAAAAAGAGGACTCGGAGGAATTCCAAACCAGTAATGACAAGGTTGAAATTAAAACAAGGTCACCGATAGAGATTCACGCATCCCAAGTATATACTAGGGCTGTTTTCCAATTGTTCTCTGAGGAGTTAATCGATAGTCTATCTTACATGGTGAAACCTGGGGAGGACGAAAGCACCGTGCAAGTTGTACGGATGAACTCGCAAGAATCATTCCTTAGGAAGGAATATCAGGTGTCTTGTGATTTGGAAAGAGAGGAATTTTCATGCGTTTGCAAGATGTTTGAGCATAAGGGGATTCTGTGCAGCCACATCTTAAGGGTACTTGTTCAGTATGGTTTGTCAAGAATTCCAGAGAGGTATATACTGAAAAGATGGACGAAGGATGCTCGGGATACTATACCACCTCATCTACATGGGTACAAAGATGATGTCGATGCTTCACAATCGAGGAGTTACAGACATGTAATGCTGAATAGGAAGACTGTGGAGGTGGCTAAGATTGCTAACAAAGATGTGCAGACATTTAAGATGGCAATGACAGTCATGAATAAGCTTTTGGAGGACATGAAAAATCAGTTATCTTTGGATGATGGTGACAATAGTAGAGAAGCTCCGAAGCGAGCAGCTAGGCATAAATCAAGGTCTACTGTATTGACTGAAGATGGAAACGAAGATGTTGGTGGAGGTGATGAGGACAGTGAGTATGACGTGCCAAGTGAGGACGAGGGTGGAAATTTGACTGCTGATATACTTCCACCGTTGAAAAAGAGAAGCAGAGGAAGACCTAAGGTGAATAGGTATAAATCGGGTGGTGAGGTTGCCAGCCTGAAAAGAAGAAAAGAAGTAGGGATTGAGAAGAAGAACACAACCAATGAGTGTGAGTCCGTTGAAGAAGAAAACCAAGTCATTGATCATGAGGATGAAGTGCCCTTTCCTCGTAGGTTTTGCCACACATGCCACGAAGCTGGTCATAATTCGCGGACTTGTGGCCGGACATCTACGTACAAGAGAAAACTTTAG

>LOC_Os05g37540 C7

ATGGATAAACAAGTGAAGAACCACAGACCTTCTGATGGTACGGATGGATCAAGGGAAATTCTTATGGACGAGCGCAACCCTACCATGGACATAGTAGATGGTATTAGTCAATCTGTCCATGATTCACAACCGGATGGTGGTCAGTCAAGCCGTGTAGCAATGGAGGAGGACGTGCCGTCGCGGGTTGGATCACATGATTCAGGGCATGGAATTCCAGAGACAAGAAATTTGGTCCCTTCACTGGATTGGACAGAAGGAAACCCATATGAAAAAGAAGGAGCTGGGATACCGATGGAAAGAATGGTGGTAGATAATGTTGATAGCAGTGCTGTCATACCTCCTGAGTATGCTGATCCTCAAATTCTTACTCCGATGCTGGGACAAAGATTTAAGACTGAGAGGGATGCCTATAATTTTTATAATGTTTATGCTGTTAGTAAAGGATTTGGGATACGACTGGACAAAGATCGCATGAATACTAAAAAGCAGAGAACAATGCGGCAAATATGTTGTTCTCACCAGGGGAGGAACCCAAAAACCAAGAAGCCTTCCGTTCGAATTGGGTGCCCAGCAATGATGAAGATAAATATGTCTGGAGCTGGTAGTGGCTGGTCAGTAACAAAGGTTGTTTCTGCGCACAACCATCCTATGAAAAAAAGTGTAGGAGTTACTAAGAATTATCAGTCGCACAATCAAATAGATGAGGGGACTCGTGGTATTATTGAAGAGATGGTAGACAGTAGTATGAGTCTTACAAATATGTATGGTATGTTGTCTGGGATGCATGGCGGACCTTCCATGGTTCCATTCACGAGAAAAGCTATGGATAGGGTTGCTTATGCAATTAGGCGGGATGAGAGCAGCGATGACATGCAAAAAACACTTGATGTCTTGAAGGATTTGCAAAAAAGGAGCAAGAATTTCTTTTATAGTATACAAGTTGACGAGGCTTGCCGTGTGAAGAACATATTTTGGTCTCATGCTGTGTCCAGATTGAACTTTGAGCATTTTGGTGATGTCATCACATTTGATACTACCTACAAAACAAACAAGTATAATATGCCCTTTGCACCATTTGTTGGTGTAAACAATCACTTTCAGAGCACCTTCTTTGGTTGTGCCCTGCTTAGAGAAGAGACTGAAGAATCATTTACATGGTTATTCAATACATTCAAAGAGTGCATGAATGGGAAGGTTCCTATTGGAATATTAACAGACAATTGTCCTTCCATGGCAGCTGCCATTAGAACAGTATTTCCCAATACAATCCATCGTGTGTGCAAGTGGCATGTACTGAAGAAAGCAAAGGAATTTATGGGGAACATATACTCCAAGCGTCATACATTCAAGAAAGCATTTCATAAGGTTCTTTGCTGGGATGACTACTACACAGAGAACTCTATGAATGGCTTTGTTAAGAGGTACGACCGGTTTTTCAATGAGAAGCTGCAAAAAGAGGACTCGGAGGAATTCCAAACCAGTAATGACAAGGTTGAAATTAAAACAAGGTCACCGATAGAGATTCACGCATCCCAAGTATATACTAGGGCTGTTTTCCAATTGTTCTCTGAGGAGTTAATCGATAGTCTATCTTACATGGTGAAACCTGGGGAGGACGAAAGCACCGTGCAAGTTGTACGGATGAACTCGCAAGAATCATTCCTTAGGAAGGAATATCAGGTGTCTTGTGATTTGGAAAGAGAGGAATTTTCATGCGTTTGCAAGATGTTTGAGCATAAGGGGATTCTGTGCAGCCACATCTTAAGGGTACTTGTTCAGTATGGTTTGTCAAGAATTCCAGAGAGGTATATACTGAAAAGATGGACGAAGGATGCTCGGGATACTATACCACCTCATCTACATGGGTACAAAGATGATGTCGATGCTTCACAATCGAGGAGTTACAGACATGTAATGCTGAATAGGAAGACTGTGGAGGTGGCTAAGATTGCTAACAAAGATGTGCAGACATTTAAGATGGCAATGACAGTCATGAATAAGCTTTTGGAGGACATGAAAAATCAGTTATCTTTGGATGATGGTGACAATAGTAGAGAAGCTCCGAAGCGAGCAGCTAGGCATAAATCAAGGTCTACTGTATTGACTGAAGATGGAAACGAAGATGTTGGTGGAGGTGATGAGGACAGTGAGTATGACGTGCCAAGTGAGGACGAGGGTGGAAATTTGACTGCTGATATACTTCCACCGTTGAAAAAGAGAAGCAGAGGAAGACCTAAGGTGAATAGGTATAAATCGGGTGGTGAGGTTGCCAGCCTGAAAAGAAGAAAAGAAGTAGGGATTGAGAAGAAGAACACAACCAATGAGTGTGAGTCCGTTGAAGAAGAAAACCAAGTCATTGATCATGAGGATGAAGTGCCCTTTCCTCGTAGGTTTTGCCACACATGCCACGAAGCTGGTCATAATTCGCGGACTTGTGGCCGGACATCTACGTACAAGAGAAAACTTTAG

>LOC_Os05g37540 HBK

ATGGATAAACAAGTGAAGAACCACAGACCTTCTGATGGTACGGATGGATCAAGGGAAATTCTTATGGACGAGCGCAACCCTACCATGGACATAGTAGATGGTATTAGTCAATCTGTCCATGATTCACAACCGGATGGTGGTCAGTCAAGCCGTGTAGCAATGGAGGAGGACGTGCCGTCGCGGGTTGGATCACATGATTCAGGGCATGGAATTCCAGAGACAAGAAATTTGGTCCCTTCACTGGATTGGACAGAAGGAAACCCATATGAAAAAGAAGGAGCTGGGATACCGATGGAAAGAATGGTGGTAGATAATGTTGATAGCAGTGCTGTCATACCTCCTGAGTATGCTGATCCTCAAATTCTTACTCCGATGCTGGGACAAAGATTTAAGACTGAGAGGGATGCCTATAATTTTTATAATGTTTATGCTGTTAGTAAAGGATTTGGGATACGACTGGACAAAGATCGCATGAATACTAAAAAGCAGAGAACAATGCGGCAAATATGTTGTTCTCACCAGGGGAGGAACCCAAAAACCAAGAAGCCTTCCGTTCGAATTGGGTGCCCAGCAATGATGAAGATAAATATGTCTGGAGCTGGTAGTGGCTGGTCAGTAACAAAGGTTGTTTCTGCGCACAACCATCCTATGAAAAAAAGTGTAGGAGTTACTAAGAATTATCAGTCGCACAATCAAATAGATGAGGGGACTCGTGGTATTATTGAAGAGATGGTAGACAGTAGTATGAGTCTTACAAATATGTATGGTATGTTGTCTGGGATGCATGGCGGACCTTCCATGGTTCCATTCACGAGAAAAGCTATGGATAGGGTTGCTTATGCAATTAGGCGGGATGAGAGCAGCGATGACATGCAAAAAACACTTGATGTCTTGAAGGATTTGCAAAAAAGGAGCAAGAATTTCTTTTATAGTATACAAGTTGACGAGGCTTGCCGTGTGAAGAACATATTTTGGTCTCATGCTGTGTCCAGATTGAACTTTGAGCATTTTGGTGATGTCATCACATTTGATACTACCTACAAAACAAACAAGTATAATATGCCCTTTGCACCATTTGTTGGTGTAAACAATCACTTTTAGAGCACCTTCTTTGGTTGTGCCCTGCTTAGAGAAGAGACTGAAGAATCATTTACATGGTTATTCAATACATTCAAAGAGTGCATGAATGGGAAGGTTCCTATTGGAATATTAACAGACAATTGTCCTTCCATGGCAGCTGCCATTAGAACAGTATTTCCCAATACAATCCATCGTGTGTGCAAGTGGCATGTACTGAAGAAAGCAAAGGAATTTATGGGGAACATATACTCCAAGCGTCATACATTCAAGAAAGCATTTCATAAGGTTCTTTGCTGGGATGACTACTACACAGAGAACTCTATGAATGGCTTTGTTAAGAGGTACGACCGGTTTTTCAATGAGAAGCTGCAAAAAGAGGACTCGGAGGAATTCCAAACCAGTAATGACAAGGTTGAAATTAAAACAAGGTCACCGATAGAGATTCACGCATCCCAAGTATATACTAGGGCTGTTTTCCAATTGTTCTCTGAGGAGTTAATCGATAGTCTATCTTACATGGTGAAACCTGGGGAGGACGAAAGCACCGTGCAAGTTGTACGGATGAACTCGCAAGAATCATTCCTTAGGAAGGAATATCAGGTGTCTTGTGATTTGGAAAGAGAGGAATTTTCATGCGTTTGCAAGATGTTTGAGCATAAGGGGATTCTGTGCAGCCACATCTTAAGGGTACTTGTTCAGTATGGTTTGTCAAGAATTCCAGAGAGGTATATACTGAAAAGATGGACGAAGGATGCTCGGGATACTATACCACCTCATCTACATGGGTACAAAGATGATGTCGATGCTTCACAATCGAGGAGTTACAGACATGTAATGCTGAATAGGAAGACTGTGGAGGTGGCTAAGATTGCTAACAAAGATGTGCAGACATTTAAGATGGCAATGACAGTCATGAATAAGCTTTTGGAGGACATGAAAAATCAGTTATCTTTGGATGATGGTGACAATAGTAGAGAAGCTCCGAAGCGAGCAGCTAGGCATAAATCAAGGTCTACTGTATTGACTGAAGATGGAAACGAAGATGTTGGTGGAGGTGATGAGGACAGTGAGTATGACGTGCCAAGTGAGGACGAGGGTGGAAATTTGACTGCTGATATACTTCCACCGTTGAAAAAGAGAAGCAGAGGAAGACCTAAGGTGAATAGGTATAAATCGGGTGGTGAGGTTGCCAGCCTGAAAAGAAGAAAAGAAGTAGGGATTGAGAAGAAGAACACAACCAATGAGTGTGAGTCCGTTGAAGAAGAAAACCAAGTCATTGATCATGAGGATGAAGTGCCCTTTCCTCGTAGGTTTTGCCACACATGCCACGAAGCTGGTCATAATTCGCGGACTTGCGGCCGGACATCTACGTACAAGAGAAAACTTTAG
