# Supplementary material for: Fine mapping of the panicle length QTL qPL5 in rice
Source: Mol Breed. 2024 Jan 17;44(2):6. doi: 10.1007/s11032-024-01443-2 (PMC10794681; doi:10.1007/s11032-024-01443-2)
Supplement: Supplementary file 3 — ESM 3 [file 11032_2024_1443_MOESM3_ESM.docx]

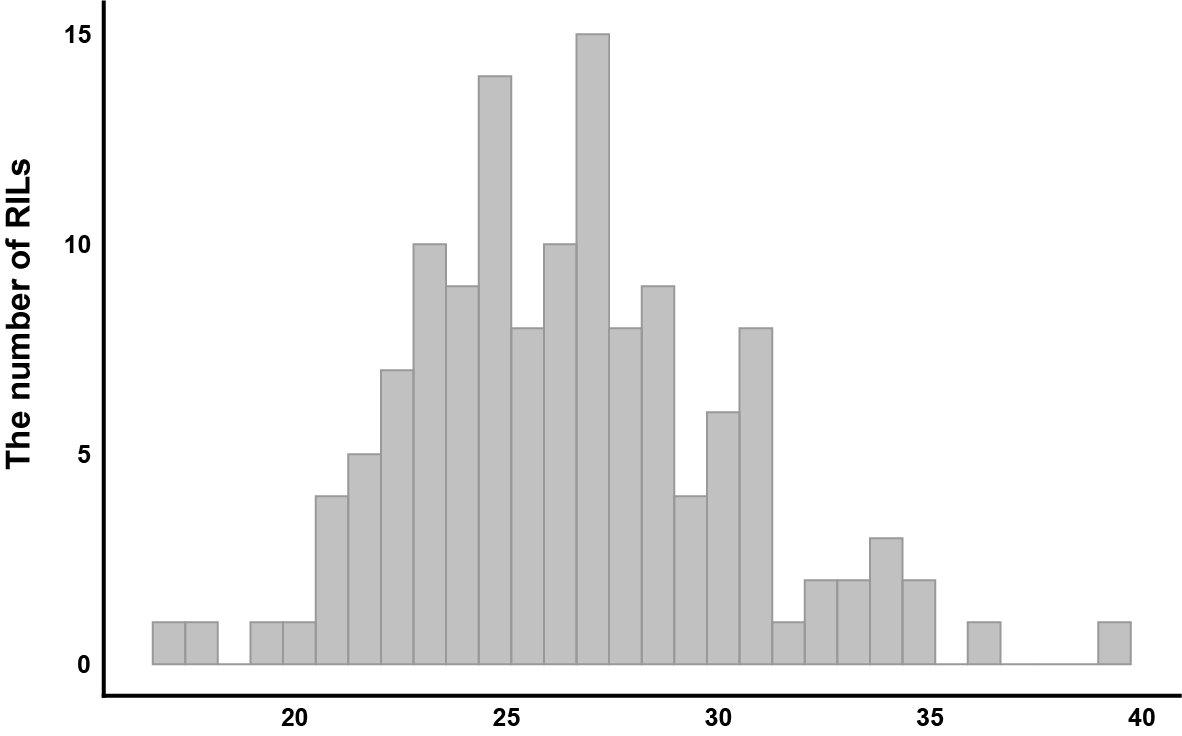


**Supplementary Fig. 1** The distribution of panicle length in the RIL population


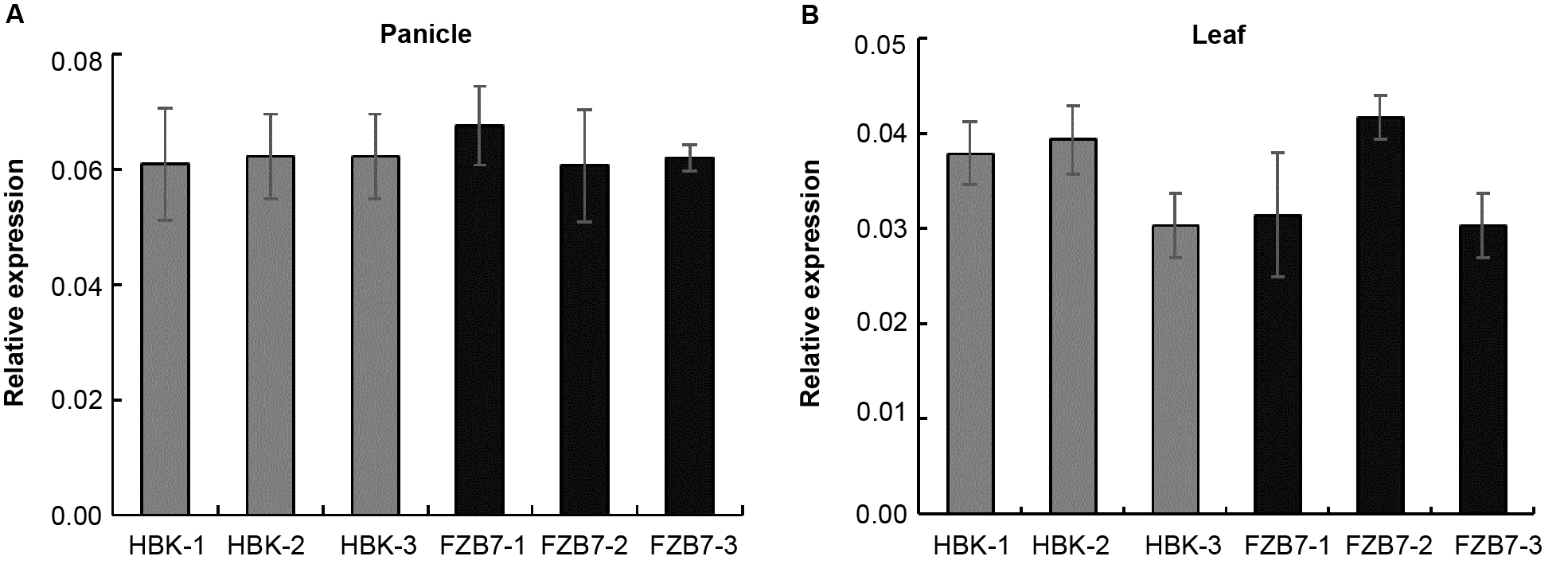


**Supplementary Fig. 2** The expression levels of *EUI1* in panicle and leaf between HBK and FZB7


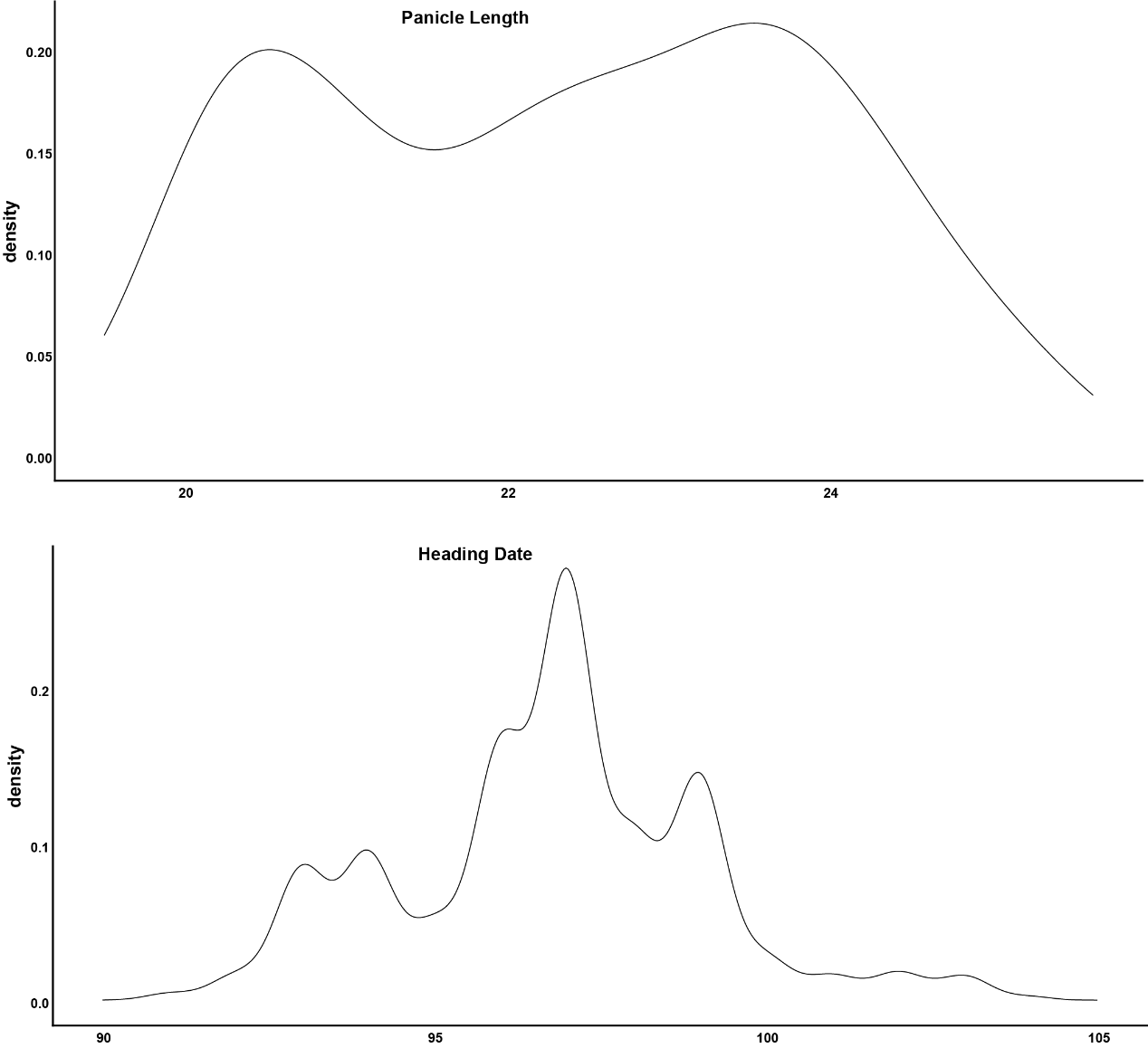


**Supplementary Fig. 3** The distribution of panicle length and heading date in the BC_4_F_2_ population.


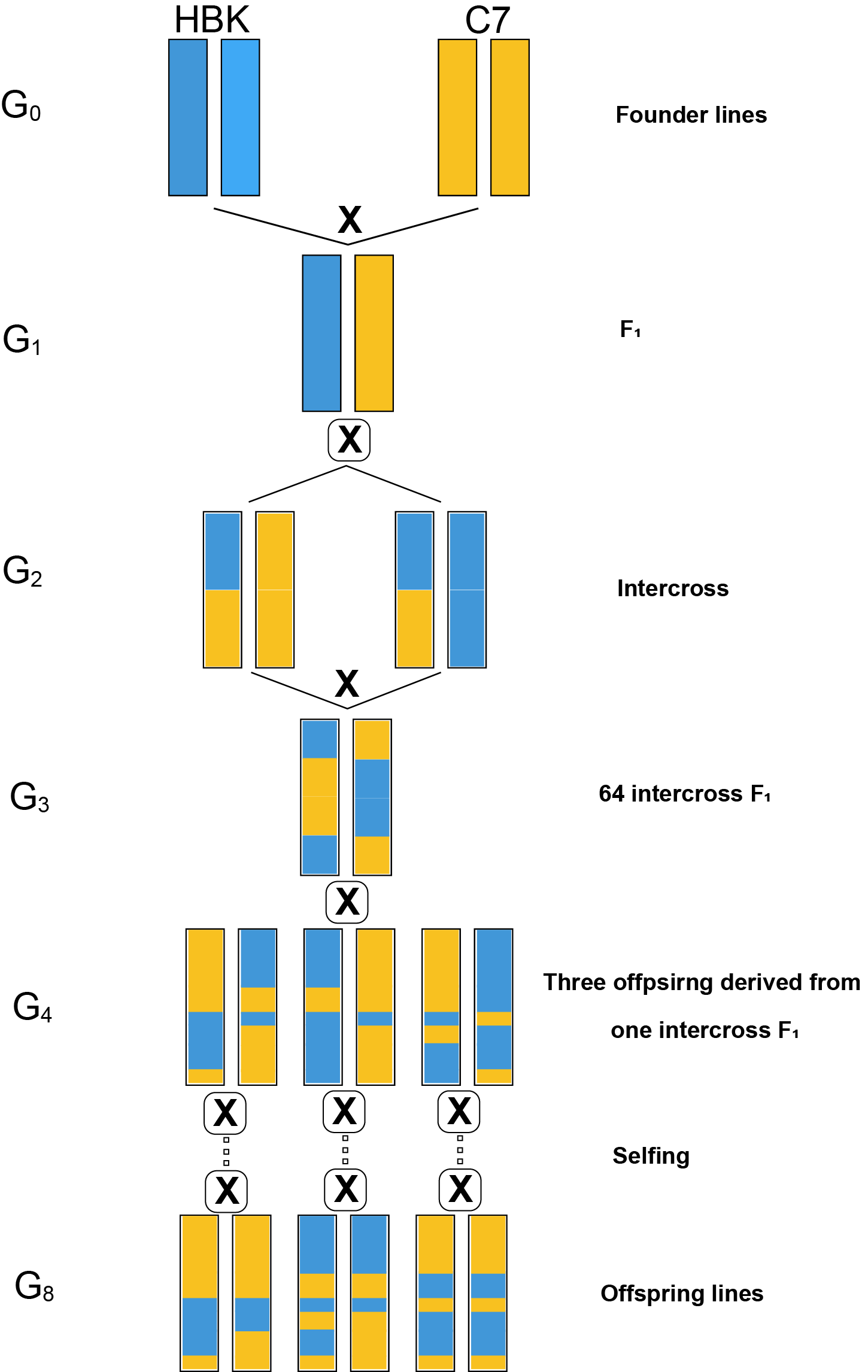


**Supplementary Fig. 4 Construction of the RIL population**
